# Retraction Note: Exploring the role of TESOL and digital technology in attitudinal change and sustainable learning for students of higher education

**DOI:** 10.1186/s40359-025-02874-y

**Published:** 2025-05-21

**Authors:** Fu Chen, Yanhong Gao, Xin Wang

**Affiliations:** College of Arts and Sciences, Northeast Agricultural University, Harbin, Heilongjiang, 150030 China

**Retraction Note: BMC Psychology (2023) 11:320**

10.1186/s40359-023-01372-3

The editor and the publisher have retracted this article. The article was submitted to be part of a guest-edited issue. An investigation by the publisher found a number of articles, including this one, with a number of concerns, including but not limited to compromised editorial handling and peer review process, inappropriate or irrelevant references or not being in scope of the journal or guest-edited issue. Based on the investigation’s findings the editor therefore no longer has confidence in the results and conclusions of this article.

Auithor Xin Wang has not explicitly stated whether they agree or disagree with the retraction. The other authors have not responded to correspondence regarding this retraction.

